# Cytotoxicity of the Sesquiterpene Lactones, Ivalin and Parthenolide in Murine Muscle Cell Lines and Their Effect on Desmin, a Cytoskeletal Intermediate Filament

**DOI:** 10.3390/toxins12070459

**Published:** 2020-07-18

**Authors:** Christo J. Botha, Y. Zethu Mathe, Gezina C. H. Ferreira, E. Annette Venter

**Affiliations:** Department of Paraclinical Sciences, Faculty of Veterinary Science, University of Pretoria, Private Bag X 04, Onderstepoort 0110, South Africa; matheyvette@gmail.com (Y.Z.M.); arina.ferreira@up.ac.za (G.C.H.F.); annette.venter@up.ac.za (E.A.V.)

**Keywords:** C2C12 (mouse skeletal myoblasts), cytoskeleton, cytotoxicity, desmin, *Geigeria*, H9c2 (rat embryonic cardiac myocytes), intermediate filament, ivalin, parthenolide, sesquiterpene lactones

## Abstract

Vermeersiekte or “vomiting disease” is an economically important disease of ruminants following ingestion of *Geigeria* (*G.*) species in South Africa. Sheep are more susceptible, and poisoning is characterized by stiffness, regurgitation, bloat, paresis, and paralysis. Various sesquiterpene lactones have been implicated as the cause of poisoning. The in vitro cytotoxicity of two sesquiterpene lactones, namely, ivalin (purified from *Geigeria aspera*) and parthenolide (a commercially available sesquiterpene lactone), were compared using mouse skeletal myoblast (C2C12) and rat embryonic cardiac myocyte (H9c2) cell lines, representing the oesophageal, skeletal and cardiac muscles, which are affected in sheep. For 24, 48, and 72 h, both cell lines were exposed. A colorimetric viability assay, 3-(4,5-dimethylthiazol-2-yl)-2,5-diphenyltetrazolium bromide (MTT), was used to assess cytotoxicity. A concentration-dependent cytotoxic response was observed in both cell lines, however, the C2C12 cells were more sensitive, with the half-maximal effective concentrations (EC_50_s) ranging between 2.7 and 3.3 µM. In addition, the effect that ivalin and parthenolide has on desmin, an important cytoskeletal intermediate filament in myocytes, was evaluated using the C2C12 myoblasts. Disorganization and aggregation of desmin were caused by both sesquiterpene lactones, which could clarify some of the ultrastructural lesions described in vermeersiekte.

## 1. Introduction

The plant poisoning vermeersiekte, also referred to as “vomiting disease”, in South Africa is caused by several *Geigeria* (*G*.) species [1,2]. Ruminants, mainly sheep, are affected after ingestion of sufficient quantities of *Geigeria* plant material [1,2]. The genus *Geigeria* is a member of the Asteraceae family and various species, such as *G. aspera*, *G. ornativa*, *G. burkei,* and *G. pectidea* are implicated in poisoning. Several sesquiterpene lactones, for example, vermeerin, vermeeric acid, geigerin, geigerinin, and ivalin, have been isolated from *Geigeria* species [1,3]. The sesquiterpene lactones, are believed to cause vermeersiekte [1,4].

After ingestion of plant material over a prolonged period (approximately 2–5 weeks with *G. aspera*), clinical signs are noticed [1]. Affected sheep walk with a stiff gait, fatigue sets in, and they lag behind the flock. The animals lose condition. The muzzle can be tinged with a greenish blotch, as the animal intermittently regurgitates ingesta (“vomition”). Regurgitation is ascribed to the severe oesophageal dilation and ingesta accumulates in the oesophageal lumen and cannot pass into the rumen [5]. As there is no tone in the flaccid enlarged oesophagus, the contents that have accumulated in the lumen of the oesophagus flow back through the nose and mouth. Mortalities due to vermeersiekte have been attributed to asphyxiation, foreign body pneumonia, paralysis of the respiratory centre, exhaustion, and cardiac failure [2]. It is interesting to note that in the United States of America, *Hymenoxys odorata* (bitter rubberweed) and *Helenium hoopesii* (orange sneezeweed) also contain sesquiterpene lactones and the latter is associated with a condition referred to “spewing sickness” in sheep [6,7].

At necropsy, an increased oesophageal diameter has been evident with consistent dilation, the so-called mega-oesophagus [1,8] and signs of foreign body pneumonia have been present in the apical and cardiac lobes of the lungs [1]. Microscopical evaluation of natural and experimentally induced vermeersiekte cases has revealed lesions in the skeletal, oesophageal, and cardiac muscles [5,9]. Histologically, skeletal, diaphragm, and oesophageal myofibres were atrophic, degenerated, and necrotic with vacuolization [5,9]. Ultrastructural lesions included myofibrillar degeneration and mitochondrial swelling in the myocardium, semimembranosus muscle, and oesophagus [5]. Thick myosin filaments disappeared first, resulting in the loss of the A-band, then loss of the thin actin filaments, followed by myofibrillar lysis. The Z-line was thickened, tortuous, fragmented, or formed clumps [5,9]. Van der Lugt and Van Heerden [5] also reported intertwined and disorderly masses of cytoskeletal filaments in cardiomyocytes of sheep in a trial, where “vermeersiekte” was experimentally induced.

Botha et al. [10] evaluated the effect of geigerin on desmin, an intermediate filament of the cytoskeleton. Aggregation and disorganization of desmin filaments were noticed after the exposure of mouse skeletal myoblast (C2C12) cells to increasing concentrations of geigerin [10]. It is inferred that the disorganization and aggregation of desmin filaments could play an important role in the pathogenesis of vermeersiekte [10]. Desmin, a muscle-specific intermediate filament, is a vital component of the cytoskeletal framework or the scaffold that maintains cell structure [11]. In addition, desmin plays an important role in cell functions [12]. Desminopathies in humans, caused by desmin and other gene mutations with a loss of desmin function, is a group of myofibrillar myopathies [11,12]. These are characterized by the presence of desmin aggregation and degenerative changes of the myofibrils and are associated with progressive skeletal myopathy and cardiomyopathy [11,12,13].

**Figure 1:**
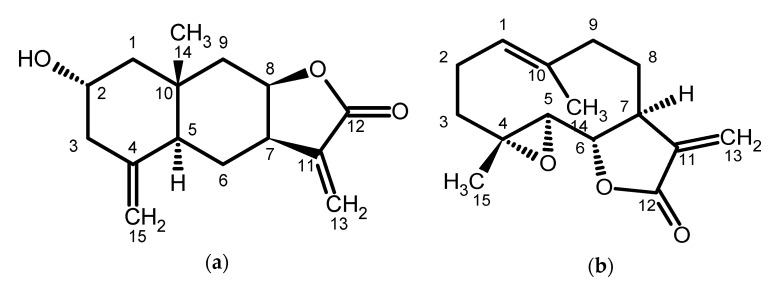
Chemical structures of (**a**) ivalin [14] and (**b**) parthenolide.

Three sesquiterpene lactones, isogeigerin acetate, ivalin, and geigerin, were recently isolated and purified from *G. aspera* [14]. The cytotoxicity of geigerin, ivalin (Figure 1a) and isogeigerin acetate was compared by exposing C2C12 myoblasts to varying concentrations of these sesquiterpene lactones for 48 h. Preliminary results indicate that ivalin is much more toxic in vitro as compared with geigerin and isogeigerin acetate [14].

The objectives of this study were twofold. Firstly, the in vitro cytotoxicity of ivalin was compared to parthenolide (a commercially available sesquiterpene lactone, Figure 1b) in mouse C2C12 and rat embryonic cardiac myocyte (H9c2) cell lines, representing the oesophageal, skeletal, and cardiac muscles affected in sheep. Secondly, immunocytochemical staining was utilized to evaluate the effect of ivalin and parthenolide on desmin intermediate filaments in the C2C12 cell line.

## 2. Results

### 2.1. In Vitro Cytotoxicity

#### 2.1.1. Cytotoxicity of Ivalin and Parthenolide in C2C12 Cell Lines

**Figure 2:**
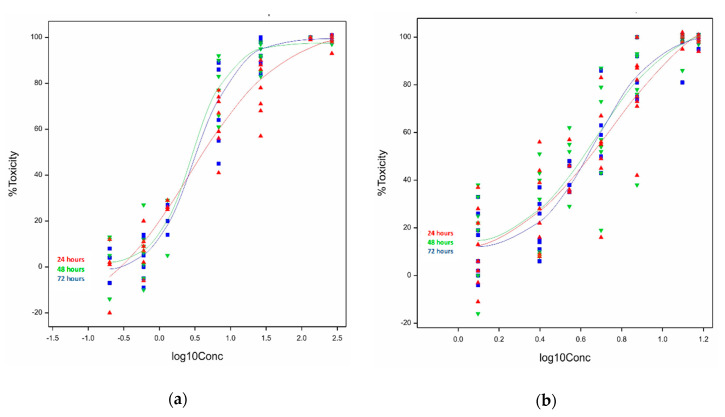
Logistic curves of the observed and fitted relationship following exposure of the mouse skeletal myoblasts (C2C12) to (**a**) ivalin and (**b**) parthenolide for 24, 48, and 72 h.

Semilogarithmic concentration response plots of C2C12 myoblasts exposed to ivalin (Figure 2a) showed that the logistic curves fitted at 48 and 72 h were similar but varied from the 24 h curve, as indicated by the slope of the curve. The slopes of the curves differed significantly (*p* = 0.014) between the incubation times, the difference was more pronounced at concentrations above the half-maximal effective concentration (EC_50_). The overall logistic fit between percentage toxicity (Y) and log concentration of ivalin (X) was highly significant (*p* < 0.001) for all the different exposure times. The minimum percentage toxicities of the curves differed significantly (*p* = 0.039), whereas no significant difference (*p* = 0.124) in the maximum percentage toxicities was observed. The EC_50_s ranged from 2.7 to 3.3 µM and are presented in Table 1. A concentration-dependent cytotoxic response of ivalin was observed.

The exposure of C2C12 cells to parthenolide showed that the logistic fit (Figure 2b) between the percentage toxicity and log concentration was highly significant (*p* < 0.001). However, none of the other parameters, i.e., the slope, and minimum and maximum toxicities differed significantly (*p* > 0.05), at all of the exposure times. The half-maximal effective concentrations (EC_50_s) of parthenolide were similar after 48 h (4.8 ± 1.1 µM) and 72 h (4.7 ± 1.1 µM). At 24 h, the EC_50_ was 5.6 (±1.2) µM (Table 1).

#### 2.1.2. Cytotoxicity of Ivalin and Parthenolide in H9c2 Cell Lines

**Figure 3:**
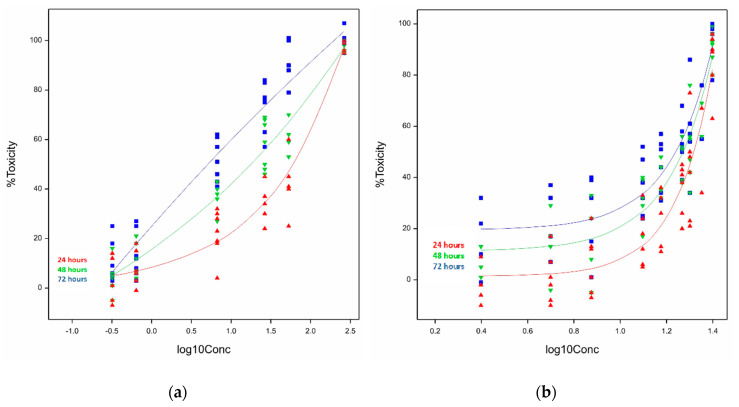
Exponential curves of the observed and fitted relationship following exposure of rat embryonic cardiac myocytes (H9c2) to (**a**) ivalin and (**b**) parthenolide for 24, 48, and 72 h.

The exponential curves of ivalin fitted at 24, 48, and 72 h showed a highly significant difference (*p* < 0.05) in terms of the minimum percentage toxicity and the shape of the curves, as can be seen from the graph (Figure 3a), but not in terms of the rate of increase (*p* > 0.05). The EC_50_s of ivalin calculated after each exposure period were tabulated (Table 1). A concentration-dependent cytotoxic response was observed.

The three curves of parthenolide fitted at 24, 48, and 72 h differed significantly (*p* < 0.05) in terms of the minimum percentage toxicity, but not in rate of increase (*p* > 0.05) and shape (*p* > 0.05) (Figure 3b). The cytotoxicity of parthenolide increased in a dose-dependent manner. The EC_50_s determined after exposure are presented in Table 1. At exposure times 48 and 72 h, the curves were the most similar.

### 2.2. Desmin Immunocytochemistry

**Figure 4:**
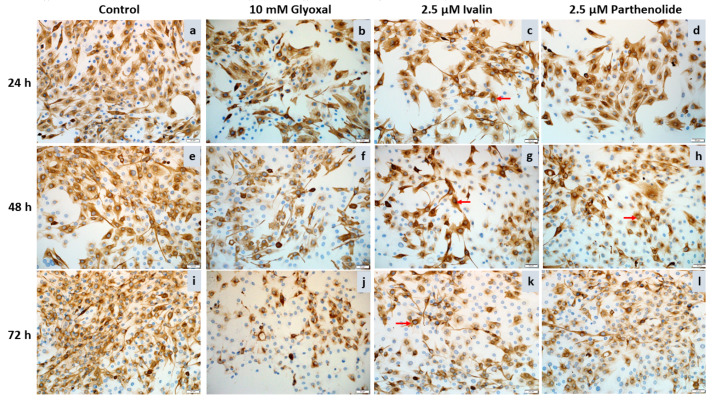
Desmin immunocytochemistry of negative control C2C12 myoblasts (**a**,**e**,**i**) and following exposure of C2C12 myoblasts to 2.5 µM ivalin (**c**,**g**,**k**) and parthenolide (**d**,**h**,**l**) and 10 mM glyoxal (positive control; **b**,**f**,**j**) for 24, 48, and 72 h. 20 × (bar = 50 µm). Perinuclear aggregation of desmin (red arrows).

Desmin, positively labeled as a shade of brown, was uniformly distributed throughout the cytoplasm of the negative control myoblasts (Figure 4a,e,i). Following ivalin and parthenolide exposure of the C2C12 myoblasts for 24, 48, and 72 h, the desmin labeling became more concentrated or aggregated, especially around the nuclei (Figure 4c,g,h,k, red arrows). In addition, over time there was an overall disorganization and decrease, even loss, of desmin intermediate filaments. The decrease in desmin filaments was clearly visible at 72 h (Figure 4k,l), especially as compared with the negative control cells (Figure 4i). There was also a decrease in desmin labeling of cells exposed to glyoxal, a positive control, over the exposure period (Figure 4b,f,j).

**Figure 5:**
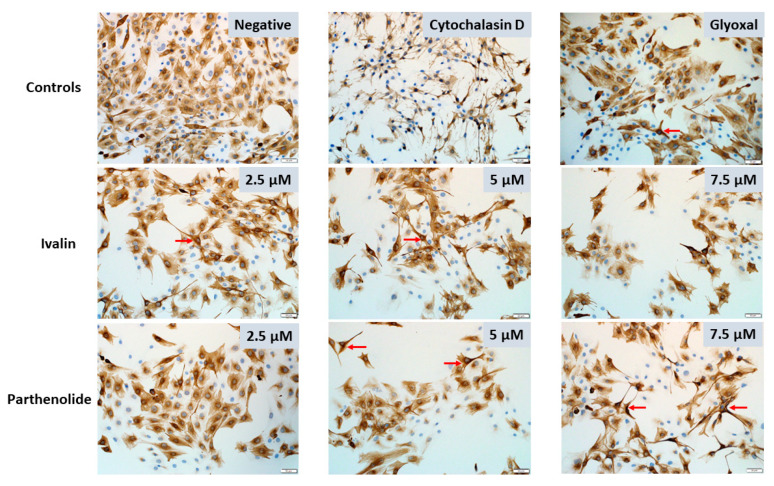
Desmin immunocytochemistry following exposure of C2C12 myoblasts to 2.5, 5, and 7.5 µM ivalin and parthenolide, for 24 h. Condensed, contracted desmin forming stellate-like cells (red arrows). Cytochalasin D (20 µM) and glyoxal (10 mM) were used as positive controls. 20 × (bar = 50 µm).

There was also a noticeable decrease in the number of myoblasts after exposure to 2.5, 5, and 7.5 µM ivalin and parthenolide after all exposure times, however, further studies are required to investigate if the decrease was also due to apoptosis as observed in C2C12 myoblasts exposed to geigerin [4]. Furthermore, following exposure of the myoblasts to ivalin and parthenolide there was a general increase in stellate-like cells with thin, elongated cytoplasm, which appeared to be similar to myoblasts exposed to glyoxal, used as positive control (Figure 5, red arrows). Desmin staining of the C2C12 cells exposed to cytochalasin D exhibited a stringy, thread-like appearance (Figure 5).

## 3. Discussion

The in vitro cytotoxicity of ivalin and parthenolide exhibited different toxic effects in C2C12 and H9c2 cells. In C2C12 myoblasts, ivalin was slightly more toxic (EC_50_s ranging from 2.7 to 3.3 µM) as compared with parthenolide (EC_50_s ranging from 4.7 to 5.6 µM) (Table 1). However, taking the standard error into account, no increasing or decreasing trend in susceptibility of the C2C12 cells over the exposure periods could be discerned. The cytotoxicity of these two sesquiterpene lactones, when exposed to C2C12 cells, were approximately 1000 times more toxic as compared with geigerin, another sequiterpene lactone purified from *G. aspera*. Fouché et al. [14] determined an EC_50_ of 3.8 mM for geigerin in C2C12 cells, after 48 h exposure. This observation was corroborated by the in vitro study conducted by Botha et al. [4] in which high geigerin concentrations (2–5 mM) were required to induce cytotoxicity in the C2C12 cell line, which was attributed to the cumulative effect of sesquiterpene lactones associated with vermeersiekte. According to Rodriguez et al. [15] the enhanced toxicity of ivalin and parthenolide can be ascribed to the presence of an α-methylene-γ-lactone group present in both sesquiterpene lactones at C-11. Ivalin also has a second exocyclic methylene group at C-4 (Figure 1a), which enhances its toxicity even further [16].

The sensitivity of H9c2 cells following ivalin and parthenolide exposure increased with an increase in exposure time (Table 1). The increase in sensitivity of H9c2 cells for ivalin between 24 and 72 h was seven times, indicating that exposure time played a major role in the susceptibility of H9c2 cardiomyocytes to ivalin. Although the increase in susceptibility of H9c2 cells to parthenolide, over time, was not as noticeable as compared with ivalin, based on the statistical ranges of the EC_50_s, there was a definite trend (Table 1). The lower EC_50_ of 20.8 µM determined for parthenolide in H9c2 cells after 24 h exposure in this study was indicative of a higher toxicity as compared with ivalin (EC_50_ of 60.7 µM). However, the EC_50_ of parthenolide compared well with a cytotoxic effect of about 65% reported by Tsai et al. [17], after 15 h exposure to 30 µM parthenolide, although 1.5× more H9c2 cells were used in their study.

In the current study, the semilogarithmic concentration-response curves fitted, to depict the relationship between cytotoxicity and concentration, were highly significant indicating that the best models were chosen to reflect the biological dose response (Figure 2 and Figure 3). The relationship between the cytotoxicity induced in C2C12 cells and toxin concentration for ivalin and parthenolide displayed the typical biological sigmoidal dose-response curve. The cytotoxic effects ranged from ≤15% for the minimum effect to 99% for the maximum effect. The statistical difference of the cytotoxic effects at the lowest concentrations tested between incubation time within cell lines was significant for ivalin (*p* = 0.039) in C2C12 cells and ivalin (*p* < 0.05) and parthenolide (*p* < 0.05) in H9c2 cells, showing that there is a positive relationship between exposure time and cytotoxicity caused at low concentrations.

The plot of the cytotoxicity of ivalin in H9c2 cells (Figure 2a) indicated a significant difference (*p* < 0.05) in the shape of the curves, however, the difference in the rate of increase of the toxic effect was not significant (*p* > 0.05). This probably could be ascribed to the variation in the biological repeats. The trends of plots of parthenolide exposure in H9c2 cells (Figure 3b) look similar and this observation was supported by the fact that the rate of increase in cytotoxicity and the shape of the curves did not differ significantly (*p* > 0.05). The significant difference (*p* < 0.05) between exposure time and toxic effect at the lowest concentration, therefore, diminished with an increase in concentration.

The cytotoxic effects in terms of basal cellular viability were evaluated using the 3-(4,5-dimethylthiazol-2-yl)-2,5-diphenyltetrazolium bromide (MTT) viability assay, thus, mitochondrial activity was used as a cytotoxicity endpoint in this study. Sesquiterpene lactones are known inhibitors of mitochondrial respiration [17,18,19], and thus the MTT assay for monitoring mitochondrial activity is a sensitive tool to study the cytotoxic effect of sesquiterpene lactones in vitro. 

The ivalin and parthenolide-induced disorganization, aggregation, and decrease of desmin intermediate filaments observed in the current study (Figure 4 and Figure 5), corroborated the previous findings where geigerin exposure of C2C12 cells caused similar effects [10]. The effect that ivalin and geigerin have on desmin could explain some of the striated muscle lesions observed in vermeersiekte. Because desmin is concentrated at the Z-lines of sarcomeres [20], the dysfunctional desmin intermediate filaments could explain some of the Z-line abnormalities (e.g., thickening, zigzagging, fragmentation, and clumping) observed ultrastructurally in *Geigeria*-poisoned sheep [5,9]. In a review of desminopathies in humans, Clemen et al. [12] conveyed reports of irregularities and other Z-line changes (streaming, loss, and rods) in skeletal muscle, as well as abnormally shaped intercalated discs (connecting cardiac muscle fibers) with convoluted and zigzag patterns. Van der Lugt and Van Heerden [5] also reported dissociation and fragmentation of intercalated discs in sheep cardiac muscle with vermeersiekte.

Furthermore, as desmin connects the contractile apparatus to the costameres [20], the observed aggregation and disorganization of desmin filaments, as revealed by immunocytochemistry in this study, could conceivably explain the disorientation and misalignment of the myofilaments and loss of myofibrils observed in experimentally induced cases of vermeersiekte in sheep [5,9]. In desminopathies in humans, also referred to as myofibrillar myopathies, degenerative changes of the myofibrillar apparatus with lysis of myofibrils are reported [12].

Glyoxal, a cellular advanced glycation end product (AGE), as well as the mycotoxin, cytochalasin D (a known actin polymerization inhibitor) were used as positive controls [13]. The alterations in the desmin filaments observed in the C2C12 myoblasts in the current study, when exposed to glyoxal or cytochalasin D, corroborated the finding by Diguet et al. [13] that both compounds contributed to the disorganization of desmin intermediate filaments.

## 4. Conclusions

In conclusion, the greater sensitivity of both C2C12 and H9c2 cell lines to ivalin at low micromolar concentrations as compared with geigerin at low millimolar concentrations provides some support for the belief that the exocyclic methylene groups enhance the toxicity of these sesquiterpene lactones [15]. A concentration-dependent cytotoxic response was observed in both cell lines, however, the C2C12 cells were more sensitive.

The sesquiterpene lactones evaluated in this study, as well as geigerin used in a previous study [4] affects desmin intermediate filaments in vitro. Possibly, in vitro exposure of muscle cell lines to sesquiterpene lactones could be used in the initial screening and experimentation to elucidate aspects of desmin-related myopathies in humans or the quest to find potential and more targeted therapies. This could possibly circumvent the exorbitant costs of using transgenic, knockout or knockin mouse models in preliminary studies.

## 5. Materials and Methods 

### 5.1. Plant-Derived Sesquiterpene Lactones

*Geigeria aspera* was collected during January 2017, in the Vrede district (27°25′48″ S; 29° 9′36″ E), Free State Province, Republic of South Africa. A voucher specimen (PRU 123161) has been lodged in the H.G.W.J. Schweickerdt Herbarium of the University of Pretoria. The plant material was dried and milled. The known sesquiterpene lactone, ivalin (Figure 1a), among other compounds, were isolated and purified [14]. Following purification, ivalin was stored in a dried form, in a dark, locked, safe deposit at room temperature. Parthenolide (Figure 1b), a commercially available sesquiterpene lactone, was purchased from Sigma-Aldrich (Darmstadt, Germany). It had been isolated and purified from the feverfew, *Tanacetum parthenium*.

### 5.2. Cell Culture

#### 5.2.1. Chemicals, Reagents, and Plastic Ware

All chemicals, reagents, and cell culture media were sourced from Sigma-Aldrich, Darmstadt, Germany, unless otherwise stated. Dulbecco’s modified Eagle’s medium (DMEM) was obtained from Pan Biotech (Aidenbach, Germany), fetal bovine serum (FBS) from Gibco (Thermo Fischer Scientific, Waltham, MA, USA), and L-glutamine and penicillin/streptomycin from Lonza (Basel, Switzerland). Cell culture flasks and microplates were acquired from Nunc (Roskilde, Denmark). Phosphate buffer saline (PBS), diethyl ether, acetonitrile, dimethyl sulfoxide (DMSO), and acetone were purchased from Merck (Darmstadt, Germany).

#### 5.2.2. Cell Lines 

Mouse skeletal myoblast (C2C12^®^ [CRL-1772^TM^]) and rat embryonic cardiac myocyte (H9c2 (2-1)^®^ [CRL-1446^TM^]) cells were purchased from the American Tissue Culture Collection (ATCC; Manassas, VA, USA). The cells were cultured at 37 °C in a humidified atmosphere of 5% CO_2_, 95% air (cell culture incubator, HeraCell 150, Thermo Fischer Scientific, Waltham, MA, USA) in high-glucose DMEM supplemented with 4 mM L-glutamine, 1 mM sodium pyruvate, 5% FBS, as well as 100 U/mL penicillin and 100 U/mL streptomycin (complete medium). After reaching 70–80% confluency within 3–4 days, the medium was removed, and the flasks rinsed twice with calcium and magnesium free PBS at pH 7.4. Cells were detached from the flasks with a trypsin (0.25%), ethylenediaminetetraacetic acid (EDTA) mixture at 37 °C for 5 min. The trypsinization was stopped by the addition of complete medium, before pelleting the cells by centrifugation at 130× *g* for 7 min at room temperature. The cellular pellet was resuspended in 2 mL complete medium and the viability of the cell suspension determined with the trypan blue exclusion test. The cell suspensions with a viability ≥95% were used for the exposure studies.

### 5.3. Exposure Studies

C2C12 cells were seeded in a 96-well microplate at 1500 cells/well and H9c2 cells at 10,000 cells/well, respectively, and placed in the cell culture incubator, allowing 24 h for attachment. The plant toxins were solubilized in organic solvents and serially diluted in complete incubation medium supplemented with 5% FBS. After preincubation, the incubation medium was removed and the cells exposed to 200 μL of a concentration range of ivalin and parthenolide, respectively, for 24, 48, and 72 h. Doxorubicin (100 µM), a mitochondrial function disruptor, was included as a positive control for the MTT cytotoxicity assay [21]. During exposure, the cells were maintained under controlled conditions to optimize their growth and viability. Exposure studies were performed in triplicate and repeated at least six times.

#### 5.3.1. MTT Cytotoxicity Assay 

At completion of the exposure period, the incubation medium was aspirated from the control and treated wells, followed by rinsing with 200 µL PBS. The MTT assay [22] was, then, initiated by the addition of 200 µL of DMEM and 20 µL of water soluble MTT (5 mg/mL in PBS) to each well and the incubation continued for 2 h, in the dark. After removal of the medium, the insoluble dark purple formazan crystals, indicative of viable cells, were solubilized by the addition of 100 µL DMSO per well and slowly shaken on a 96-well plate shaker (Microporous Quick Shaker QB-9001, Hinotek Group Ltd., Ningbo, China), in the dark, for 5 min. Then, viable cells were quantified in terms of the intensity of the ABS of the solubilized formazan, read at 570 nm with a reference wavelength of 630 nm using a microplate reader (HT Synergy, Biotek Instruments, Inc., Winooski, VT, USA).

#### 5.3.2. Desmin Immunocytochemistry

For immunocytochemistry, 5000 cells in 400 µL media were seeded per slide in a Lab-TEK 8-well chamber slide system and incubated at 37 °C in a humidified atmosphere of 5% CO_2_. After 24 h, the cells were exposed to 2.5, 5, and 7.5 µM ivalin and parthenolide for 24, 48, and 72 h. Untreated cells, exposed to complete media only, were used as negative controls. The positive controls were comprised of cells exposed to 10 mM glyoxal and 20 µM cytochalasin D, for 30 min before staining. Staining of the intermediate filament, desmin, was accomplished by following the methodology previously described [10]. Briefly, immunocytochemistry was performed using a manual chromogen-based indirect immunoperoxidase technique. Slides with cultured C2C12 were air-dried, then fixed in 4% buffered formalin for 30 min, followed by rinsing for 10 min in 70% ethanol. Next, slides were incubated with 3% hydrogen peroxide in methanol for 5 min, and then, heat-induced epitope retrieval (HIER) was performed by microwave heating (96 °C) of the slides in EDTA buffer (pH = 9) for 21 min. To quench non-specific background staining, slides were treated with normal horse serum (Sigma-Aldrich, Saint Louis, MO, USA) diluted 1:10 with PBS buffer (pH = 7.6) that contained 0.1% bovine serum albumin (BSA), for 20 min, at room temperature. A mouse monoclonal anti-human desmin antibody (DakoCytomation, Glostrup, Denmark) was applied to the sections (1:200 dilution for 60 min), followed by application of the EnVision Rabbit/Mouse polymer detection system (Dako, Glostrup, Denmark) according to the manufacturer’s instructions. The 3,3′-diaminobenzidine (DAB) chromogen, that was supplied with the EnVision system, was applied to the cells for 1–2 min, followed by counterstaining with Mayer’s hematoxylin for 20 s. Slides were subsequently rinsed in tap water for 10 min, routinely dehydrated, mounted with entellan, and secured with a coverslip prior to examination under the light microscope.

### 5.4. Statistical Analysis 

The percentage cytotoxicity obtained from applying different concentrations of a toxic sesquiterpene lactone is a typical dose-response experiment and requires the fitting of the best model to represent the response. This was done for each time period separately (24, 48, and 72 h) for the two different cell lines. A logistic model is the best and most widely accepted model to fit if the data follows a typical sigmoidal (S-shape) trend and for calculation of the estimated median effective concentration (EC_50_) [23]. A logistic curve was fitted for ivalin and parthenolide cytotoxicity data in C2C12 cells using the model, Y = A + C/(1 + EXP[−B*(X − M)]), where X is the different (log10) concentrations applied per toxin and cell line. The estimated regression parameter A is the minimum % toxicity, A + C is the maximum % toxicity, M is the EC_50_, and B is the maximum increase in toxicity at EC_50_.

For data following an exponential trend, i.e., the ivalin and parthenolide toxicity data in H9c2 cells, the ordinary exponential curve, also known as the asymptotic regression curve, was fitted. The model, Y = A + B × (R)^X^ represented a curve rising from an asymptote and was fitted to the different (log10) concentrations (X) applied per toxin and cell line. The estimated regression parameter A is the minimum % toxicity, R determines the shape of the curve, and B is the rate of increase. The EC_50_ was calculated from this model. Thereafter, the test of parallelism was applied to ascertain if the curves differed between times for the same cell line and at the same time between different cell lines. The estimated regression parameters (not times) were compared for the exponential curves [24]. 

Accumulated analysis of variance (ANOVA) was used to statistically interpret the overall logistic fit between Y (% toxicity) and X (logarithmic (log10) concentration of toxin) for all exposure times, as well as differences between exposure time at the lowest toxin concentrations, the highest toxin concentrations and the slope of the dose-response curve at the EC_50_. Data were analyzed using the statistical program GenStat^®^ (VSN International, Hemel Hempstead, England, UK, 2017).

Research ethics approval was obtained from the committee appointed by the Faculty of Veterinary Science, University of Pretoria (REC026-18 and REC127-19). 

## Figures and Tables

**Table 1:** Half-maximal effective concentrations (EC_50_s) of ivalin and parthenolide determined on C2C12 and H9c2 cells after 24, 48, and 72 h.

Cell Line.	Exposure Time (h)	Ivalin ^1^ (µM)	Parthenolide ^1^ (µM)
C2C12	24	2.7 ± 1.6 *n* = 7	5.6 ± 1.2 *n* = 8
	48	3.0 ± 1.2 *n* = 7	4.8 ± 1.1 *n* = 8
	72	3.3 ± 1.2 *n* = 7	4.7 ± 1.1 *n* = 8
H9c2	24	60.7 ± 1.1 *n* = 7	20.8 ± 1.0 *n* = 6
	48	17.8 ± 1.1 *n* = 7	18.4 ± 1.0 *n* = 6
	72	8.5 ± 1.3 *n* = 7	17.4 ± 1.0 *n* = 6

^1^ EC_50_ ± standard error of the mean (SE) for at least 6 experimental repeats (*n*).
